# Integrated network pharmacology and *in vivo* evidence reveal vitamin E’s multi-organ protective effects in acute lung injury and secondary enteritis

**DOI:** 10.3389/fimmu.2026.1762374

**Published:** 2026-03-18

**Authors:** Jinhai Shao, Qiongdan Zhang, Huanlong Du, Dongzhi Wang, Jiwei Mao, Guifang Gu

**Affiliations:** 1 Department of Critical Care Medicine, Shangyu People’s Hospital of Shaoxing, Shaoxing University, Shaoxing, China; 2 Department of The First Clinical Medical School, Hainan Medical University, Haikou, China; 3 Hainan Academy of Medicine Sciences, Haikou, China; 4 Department of Radiation Oncology, Shaoxing People’s Hospital, Shaoxing, China; 5 Department of Respiratory Medicine, Shangyu People’s Hospital of Shaoxing, Shaoxing University, Shaoxing, China

**Keywords:** acute lung injury, lung–gut axis, network pharmacology, secondary enteritis, vitamin E

## Abstract

**Introduction:**

Acute lung injury (ALI), characterized by diffuse alveolar damage and inflammation, can trigger secondary enteritis through systemic interactions, aligning with the established lung-gut axis concept—a bidirectional communication system underpinned by shared mechanisms including inflammation, oxidative stress, and barrier dysfunction; however, the molecular links remain unclear, and vitamin E’s role in this axis lacks systematic investigation.

**Methods:**

By integrating GeneCards database screening, protein–protein interaction (PPI) network, and pathway analyses,

**Results:**

we identified 34 “ALI–Enteritis ∩ Vitamin E” related core genes. In an LPS-induced ALI mouse model, vitamin E alleviated lung pathology while repairing intestinal mucosal barrier through modulating M1/M2 macrophage polarization and suppressing excessive neutrophil recruitment. This study first systematically delineates molecular commonalities between ALI and enteritis, identifies 34 shared therapeutic targets, and demonstrates vitamin E’s coordinated multi-organ protection via targeting these pathways,

**Discussion:**

offering novel insights into the “lung–intestine axis” and a scientific basis for its nutritional-immunomodulatory intervention in multi-organ injury.

## 1 Introduction

Acute lung injury (ALI) is a life-threatening systemic inflammatory response syndrome localized to the lung, characterized by disruption of the alveolar–capillary barrier, massive inflammatory cell infiltration, and profound oxidative stress (1). Accumulating evidence highlights a bidirectional immune crosstalk between the lung and gut mediated by immune cell trafficking (2), microbial metabolites (**1**), and inflammatory networks (3), which lays the foundation for the lung-gut axis theory. Clinically, some of influenza patients exhibit intestinal barrier dysfunction (4), and animal studies demonstrate that respiratory viral infections can drive intestinal Th17 accumulation and MUC2 dysregulation, indicating that pulmonary inflammation may perturb gut homeostasis via systemic immune activation (5). However, specific interventions for secondary enteritis during ALI remain limited, with most current therapies focusing on pulmonary support rather than coordinated gut protection.

**Figure 1:**
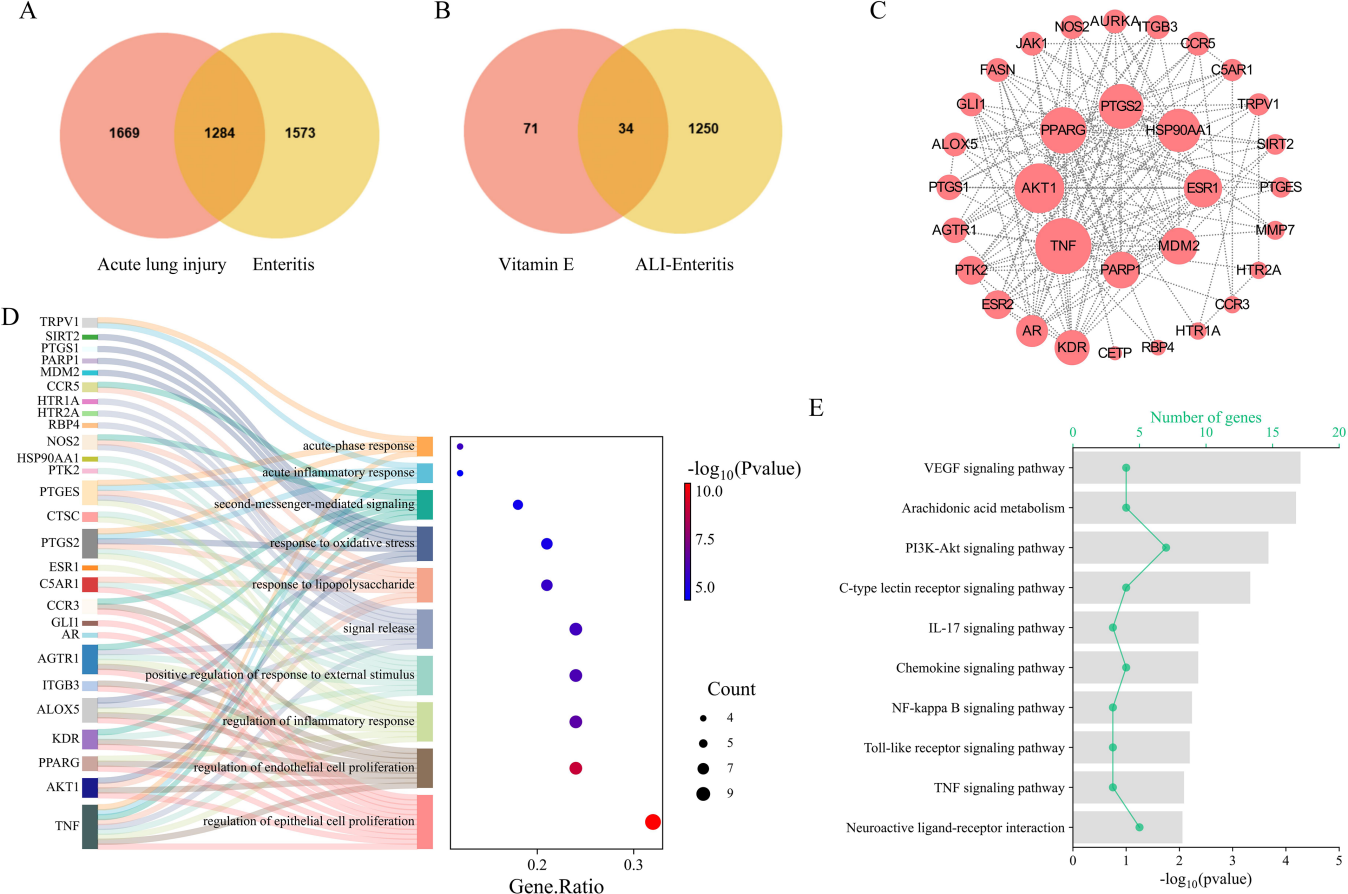
Identification of core genes and functional enrichment analysis linking ALI, enteritis, and Vitamin E: **(A)** Venn diagram showing the intersection of disease-secondary genes between acute lung injury (ALI) and enteritis; 1284 overlapping genes were identified through GeneCards database screening. **(B)** Venn diagram of the intersection between ALI–Enteritis shared genes and vitamin E target genes, resulting in 34 “ALI–Enteritis ∩ Vitamin E related” core genes. **(C)** Protein–protein interaction (PPI) network of core genes; nodes represented genes, edges represented interaction relationships, and the top 8 genes with the highest connectivity (Hub Genes) were highlighted. **(D)** GO BP enrichment analysis of core genes; the x-axis represented Gene.Ratio (ratio of core genes enriched in the pathway to total core genes), the y-axis represented enriched BP terms, and the color intensity indicated -log10(Pvalue) (higher values indicated more significant enrichment). **(E)** KEGG pathway enrichment analysis of core genes; the line represented the number of core genes enriched in the pathway, the y-axis represented enriched pathways, and the column height indicated -log10(Pvalue).

Vitamin E, an important lipid-soluble antioxidant, has α-tocopherol as its core component (6). In the body, it mainly exerts protective effects by neutralizing free radicals, inhibiting lipid peroxidation chain reactions, and regulating multiple inflammatory signaling pathways (7). Previous studies have confirmed that Vitamin E shows significant anti-inflammatory and antioxidant effects in various inflammatory diseases. For instance, in a mouse model of lipopolysaccharide (LPS)-induced acute lung injury (ALI), Vitamin E can reduce the release of pro-inflammatory factors such as TNF-α and IL-1β by inhibiting the NF-κB pathway (8); in a rat model of dextran sulfate sodium-induced ulcerative colitis, it can improve intestinal barrier function by maintaining the expression of tight junction proteins in intestinal epithelial cells (9). However, the role of Vitamin E in the complex pathological process of acute lung injury and its subsequent colitis has not been systematically studied.

In recent years, network pharmacology, as a systems biology approach that integrates drug chemical components, biological targets, and disease associations, has been able to systematically predict the multi-target synergy mechanism of drugs by constructing a “drug-target-disease” multi-dimensional network (10). Based on this, this study first screened the key target genes shared by Vitamin E and ALI/enteritis through network pharmacology, clarifying their potential functional nodes; to empirically validate these computational predictions and translate the network data into biological significance, we subsequently utilized an LPS-induced ALI animal model treated with Vitamin E to explore the following core aspects: (1) pulmonary tissue pathological damage and the level of key inflammatory factor TNF-α; (2) intestinal tissue morphology, barrier function, and inflammatory factor levels (IL-17, TNF-α); (3) the distribution of immune cell subsets in lung and colon tissues, aiming to elucidate the molecular mechanism by which Vitamin E regulates the local immune microenvironment and barrier function of the lung and colon, thereby improving the lung-gut axis, and providing theoretical basis and new ideas for the comprehensive treatment of multiple organ damage.

## 2 Methods

### 2.1 Acquisition of genes related to acute lung injury and enteritis diseases

GeneCards is a comprehensive, searchable database that integrates genomic, transcriptomic, proteomic, and clinical data from over 150 web sources. It is widely used to identify robust disease-gene associations and discover potential therapeutic targets. In our study, it assigns a comprehensive Relevance Score. This study first retrieved lists of genes significantly related to both diseases by searching keywords “Acute Lung Injury” and “Enteritis” in the GeneCards database (https://www.genecards.org/). GeneCards integrates literature mining, experimental validation, and multi-omics data, assigning a comprehensive relevance score (Relevance Score, ranging from 0 to 100) to each gene. The top 25% high-scoring genes were selected as candidate disease targets to ensure a high correlation between the selected genes and the disease phenotype. The gene sets related to ALI (GeneSet_ALI) and enteritis (GeneSet_Enteritis) were obtained, and the intersection of the two sets was taken using the Venn diagram tool to obtain the “ALI ∩ Enteritis” gene set representing the potential common pathological targets of the two diseases.

### 2.2 Acquisition of genes related to Vitamin E drug interactions

This study predicted human protein targets directly interacting with Vitamin E through the SwissTargetPrediction database (https://www.swisstargetprediction.ch/). Selecting the species as “Homo sapiens”, inputting the SMILES structure of Vitamin E (CC1=C(C(CCC1)(C)C)C(C(C(C(C)C)O)CC(C)C)O), and submitting the prediction request. The database outputs potential direct binding targets of Vitamin E based on molecular docking, pharmacophore matching, and structural similarity algorithms, representing the potential direct action targets of Vitamin E.

### 2.3 Intersection analysis of diseases and drug targets

To identify the core targets simultaneously involved in the pathological processes of ALI and enteritis and potentially regulated by Vitamin E, the “ALI ∩ Enteritis” gene set (ALI-Enteritis) was intersected with the “Vitamin E-related gene set” (Vitamin E) based on a unified “Gene Symbol”. The “ALI-Enteritis ∩ Vitamin E-related” gene set (i.e., intersecting genes simultaneously associated with ALI, enteritis, and Vitamin E) was obtained as the target pool for subsequent network analysis and functional validation.

### 2.4 Construction of protein-protein interaction network and selection of core genes

The UniProt IDs corresponding to the intersection genes of the drug and the two diseases were imported into the STRING database (https://string-db.org/, version 12.0, species selection “Homo sapiens”, confidence threshold set to 0.4), and a protein-protein interaction network was constructed. Network nodes represent the encoded proteins of genes, and the edges represent the experimentally verified or highly confident predicted interaction relationships between proteins. The PPI data output by STRING was imported into the Cytoscape software (version 3.9.1) for visualization and topological analysis. By calculating the centrality index Degree of each node, the nodes ranked from Top 1 to 8 in Degree were selected as core genes (Hub Genes). The Degree value reflects the number of direct connections of the node with other nodes and is a key indicator for evaluating the “hub position” of the protein in the network. Genes with high Degree values usually play a core regulatory role in disease-drug interaction networks.

### 2.5 Functional enrichment analysis

To analyze the biological functions and potential pathways of the intersection genes of drugs and the two diseases, the gene sets were analyzed by Gene Ontology (GO) biological process (BP) analysis and Kyoto Encyclopedia of Genes and Genomes (KEGG) pathway analysis. The GO BP analysis focused on the biological processes participated by the genes, while the KEGG analysis identified the enriched signaling pathways of the genes. Key biological processes and pathways were selected based on the enrichment results, combined with the functional annotations of the core genes, to explain the possible common pathological mechanism by which vitamin E simultaneously intervenes in ALI and enteritis through regulating which core targets and pathways.

### 2.6 Animal model construction and grouping

Healthy adult male C57BL/6 mice (6–8 weeks old, 18–22 g) were obtained from Beijing Vital River Laboratory Animal Technology Co., Ltd. (Beijing, China) and housed under SPF conditions for 1 week prior to experimentation. Animal procedures were in accordance with the Guide for the Care and Use of Laboratory Animals published by the National Institutes of Health and were approved by the Institutional Animal Research and Use Committee of Shaoxing People ‘s Hospital (No. 2025Z021). Studying the lung-gut axis using live respiratory viruses is often confounded by the pathogen’s capacity to directly infect the intestinal epithelium. Therefore, employing a sterile inflammatory trigger, such as lipopolysaccharide (LPS), provides a more controlled and precise paradigm. Mice were randomly assigned to 3 groups (n = 5/group): control, LPS model, and VE. The experimental procedure was as follows: 4 hours before the start of the experiment, all mice received intranasal instillation. The control group was instilled with normal saline, while the LPS model group and the VE group were instilled with 5 mg/kg LPS solution (LPS from Escherichia coli O55:B5, Sigma-Aldrich, St. Louis, MO, USA); the drug intervention was initiated 4 hours after LPS instillation. The control group and the LPS model group were intragastrically administered the same volume of normal saline daily, while the VE group was given 30 mg/kg/d of Vitamin E (Sigma-Aldrich, St. Louis, MO, USA); 72 hours after LPS instillation, all mice were euthanized, and lung and colon tissue samples were collected for subsequent detection and analysis to explore the intervention effect of Vitamin E in the LPS-induced acute lung injury model and related mechanisms.

### 2.7 HE Staining and quantitative histological scoring

Tissues were fixed in 4% paraformaldehyde, dehydrated through a graded ethanol series, cleared in xylene, embedded in paraffin, and sectioned (5 μm). Sections were mounted on slides, dewaxed, rehydrated, and stained with hematoxylin (nuclei, blue) for 10 minutes, differentiated with 1% hydrochloric acid-ethanol, blued with ammonia water, and counterstained with eosin (cytoplasm, red) for 5 minutes. After dehydration through graded ethanol and xylene clearing, slides were mounted with neutral resin.

Sections were stained with routine hematoxylin-eosin (HE) and then randomly imaged using a digital pathology scanning system. The degree of alveolar inflammation was assessed using a double-blind method, based on the standardized scoring system established by Matute-Bello et al (1). This system quantifies alveolar inflammation by evaluating the density of inflammatory cell infiltration and the extent of alveolar structural changes (0 = no injury, 1 = mild, 2 = moderate, 3 = severe). For colonic injury, a standardized histopathological score established by Chiu was used to quantify mucosal damage and inflammatory cell recruitment: Grade 0: normal mucosa; Grade 1: development of a sub-epithelial space at the tips of the villi; Grade 2: more extended sub-epithelial space at the tips of the villi, development of Gruenhagen’s space at the tips of the villi; Grade 3: massive epithelial lifting down the sides of the villi, villus necrosis; Grade 4: villi are denuded of epithelial layer; Grade 5: loss of villi, mucosal ulceration and necrosis with invasion of the muscularis propria (11).

### 2.8 AB-PAS staining

Sections were dewaxed, rehydrated, and sequentially stained with alcian blue (pH 2.5, acidic mucins, blue, 5–10 minutes), periodic acid (oxidized neutral mucins, 5–10 minutes), and Schiff’s reagent (neutral/acidic mixed mucins, purple-blue, 15–30 minutes in the dark). Nuclei were counterstained with hematoxylin if needed, followed by dehydration and xylene clearing. Microscopic observation distinguished acidic mucins (blue), neutral mucins (magenta), and mixed mucins (purple).

### 2.9 Colon MUC2 immunofluorescence

Colon tissues were fixed in 4% paraformaldehyde, dehydrated, cleared, and embedded in paraffin. Sections were dewaxed, rehydrated, and permeabilized with 0.1% Triton X-100 for 5 minutes. After blocking with 10% normal goat serum for 1 hour at room temperature, sections were incubated overnight at 4 °C with rabbit anti-MUC2 primary antibody (1:200 dilution; Abcam, Cambridge, UK, Cat#ab134119). Following three PBS washes, Alexa Fluor 488-conjugated goat anti-rabbit secondary antibody (1:500 dilution; Invitrogen, Thermo Fisher Scientific, Waltham, MA, USA, Cat#A-11008) was applied for 1 hour at 37 °C in the dark. Nuclei were counterstained with DAPI (4’,6-diamidino-2-phenylindole, Vector Laboratories, Burlingame, CA, USA), and slides were mounted with an anti-fade mounting medium (Beyotime Biotechnology, Shanghai, China, Cat#ab104135). Fluorescence microscopy was used to visualize MUC2 (green) and nuclei (blue). Finally, images were acquired using an Olympus VS200 Panoramic Slide Scanner.

### 2.10 Cytokine detection

Lung and colon tissue homogenates (supernatants) from the control, LPS model, and VE groups were assayed for TNF-α levels, and colon tissue homogenates (supernatants) were additionally assessed for IL-17 levels. All ELISAs were performed according to the manufacturers’ protocols using commercially available domestic kits: mouse TNF-α ELISA kit (Beyotime Biotechnology, Shanghai, China, Cat#PT512) and mouse IL-17 ELISA kit (Beyotime Biotechnology, Shanghai, China, Cat#PI545).

### 2.11 Lung and colon tissue immune cell isolation and flow cytometry analysis

After digestion of lung tissues to prepare single-cell suspensions, macrophage M1/M2 polarization analysis was performed. In the experiment, the myeloid lineage marker CD11b-FITC (BioLegend, San Diego, CA, USA) and the mature macrophage-specific marker F4/80-PE/Cyanine7 (BioLegend, San Diego, CA, USA) were used for gating the target cell population. Subsequently, the M1-type macrophage-specific marker CD86-PE (BioLegend, San Diego, CA, USA) and the M2-type macrophage-specific marker CD206-APC (BioLegend, San Diego, CA, USA) were detected to clarify the different polarization statuses of macrophages.

Colon tissues were first placed in HBSS buffer containing 2 mM EDTA and incubated at 37 °C for 15 minutes, repeated 3 times to remove epithelial cells. The remaining tissues were transferred to RPMI-1640 medium supplemented with 0.425 mg/mL collagenase V and 10% FBS, followed by digestion at 37 °C for 30–45 minutes. The digested products were sequentially filtered through 100 μm and 70 μm cell strainers, centrifuged at 300 × g for 5 minutes, and washed twice with PBS to finally prepare single-cell suspensions at a concentration of 1×10^6^ cells/100 μL PBS. CD8^+^ T cells were detected by combining the total T cell marker CD3-FITC (BioLegend, San Diego, CA, USA) and the cytotoxic T cell marker CD8-APC (BioLegend, San Diego, CA, USA); simultaneously, the neutrophil-specific marker Ly6G-APC (BioLegend, San Diego, CA, USA) and the myeloid broad marker CD11B-FITC (BioLegend, San Diego, CA, USA) were used to analyze neutrophil subsets.

For both tissues, after staining, cells were washed twice with PBS, resuspended in 300 μL PBS, and analyzed via flow cytometry (BD LSRFortessa).

### 2.12 Statistical analysis

All data are mean ± SD. The homogeneity of variances was verified by the Brown-Forsythe test. Student’s t test or ANOVA with Bonferroni multiple comparison test was used for statistical differentiation between groups, where appropriate. *p* < 0.05 was considered to indicate statistically significant difference. GraphPad Prism 9.2.0 (GraphPad software, San Diego California USA) was used for all statistical computations.

## 3 Results

### 3.1 Identification of core genes and functional enrichment analysis linking ALI, enteritis, and Vitamin E

Acute lung injury (ALI) and enteritis exhibited bidirectional interactions via the “lung–intestine axis.” Through GeneCards database screening, ALI and enteritis were found to have distinct sets of highly correlated genes, with 1284 overlapping genes (ALI–Enteritis shared gene set; **Figure 1A**), indicating extensive molecular commonalities in pathological responses such as inflammation, oxidative stress, and barrier dysfunction. Intersection analysis between the ALI–Enteritis shared gene set and vitamin E target genes identified 34 “ALI–Enteritis ∩ Vitamin E related” core genes (**Figure 1B**), which serve as potential molecular links between disease pathogenesis and vitamin E’s pharmacological effects. Protein–protein interaction (PPI) network construction for these core genes revealed top 8 Hub Genes with high connectivity (**Figure 1C**), suggesting pivotal roles in both diseases. GO BP analysis showed significant enrichment in inflammatory response and oxidative stress response (**Figure 1D**), while KEGG pathway analysis identified enrichment in TNF signaling pathway and IL–17 signaling pathway (**Figure 1E**), all of which are critical for inflammation regulation and cell fate determination.

### 3.2 Protective efficacy of vitamin E in LPS-induced ALI mouse model

**Figure 2:**
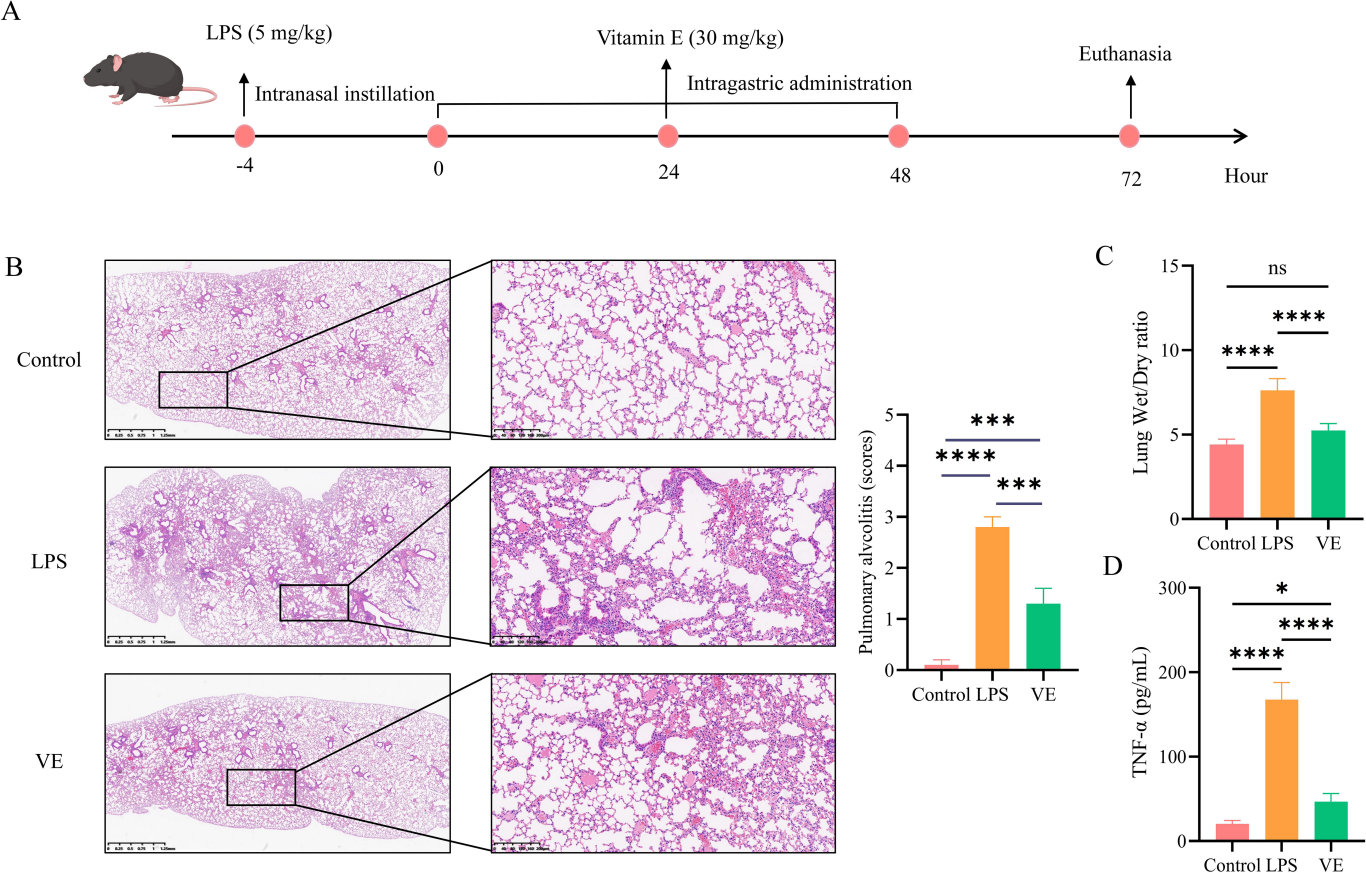
Protective effect of Vitamin E on LPS-induced ALI in mice. **(A)** Schematic diagram of the experimental design. **(B)** HE staining and injury score of lung tissue sections: panoramic images (scale bar = 1.25 mm) showing the overall distribution of lung tissue structure, and partial magnified images (scale bar = 200 μm) displaying alveolar structure, inflammatory cell infiltration, and interstitial edema in the Control, LPS and VE groups. **(C)** Lung wet/dry ratio of different treatment groups. **(D)** Bar graph showing TNF-α levels in lung tissue detected by enzyme-linked immunosorbent assay (ELISA). Data are represented as mean ± SD. *p < 0.05, **p < 0.01, ***p < 0.001,****p < 0.0001.

An LPS-induced ALI mouse model was established to verify Vitamin E’s *in vivo* efficacy (**Figure 2A**). Intranasal LPS instillation successfully induced characteristic ALI pathological changes: compared with the control group, the LPS model group showed significant inflammatory cell infiltration, alveolar structural disruption, and interstitial edema (**Figure 2B**), leading to a significant increase in the score (**Figure 2B**); the lung wet/dry ratio was markedly increased (**Figure 2C**); and lung tissue TNF-α levels were significantly elevated (**Figure 2D**). In contrast, Vitamin E treatment significantly ameliorated these pathological indices, including histopathological alterations, significant reduction in the lung injury score, lung wet/dry ratio, and TNF-α levels, confirming the reliability of the model and Vitamin E’s protective effect against LPS-induced ALI.

### 3.3 Amelioration of LPS-associated secondary enteritis by vitamin E

**Figure 3:**
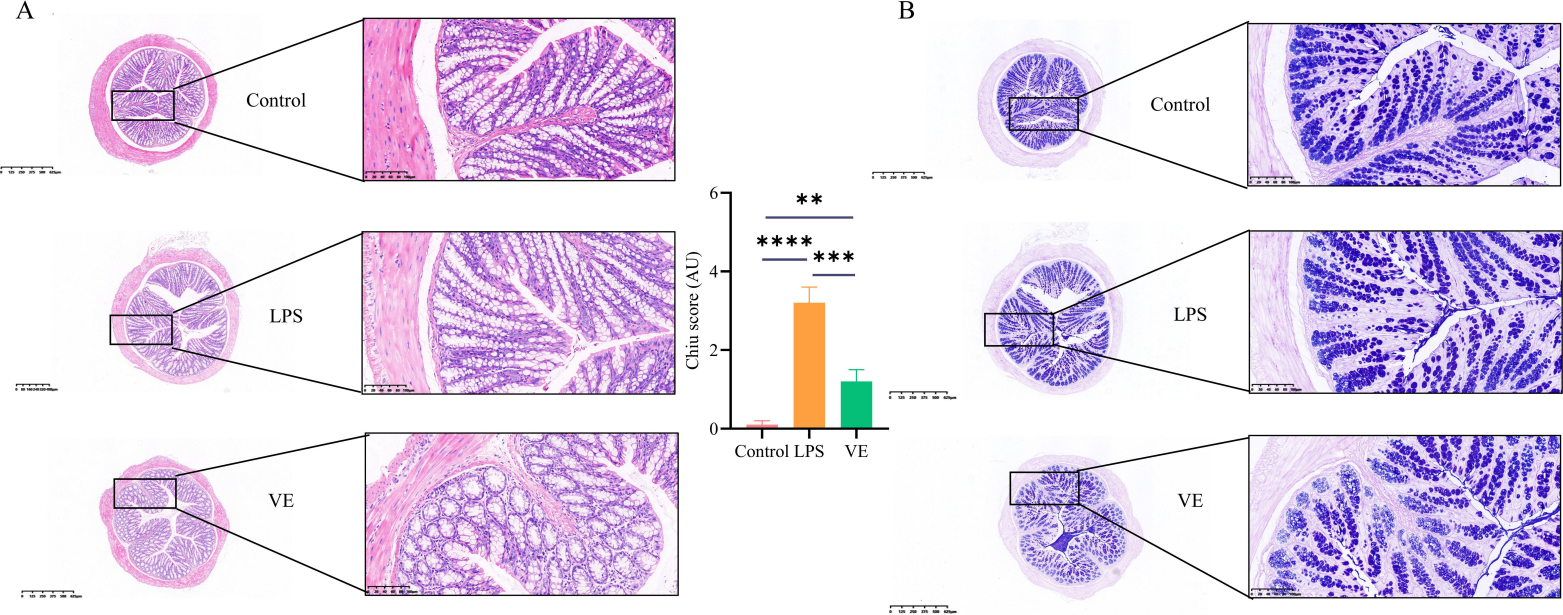
Ameliorative effect of Vitamin E on LPS-induced secondary intestinal injury. **(A)** HE staining and injury score of colon tissue sections: panoramic images (scale bar = 625 μm) showing the overall structure of colon tissue, and partial magnified images (scale bar = 100 μm) displaying mucosal epithelial integrity, glandular arrangement, and inflammatory cell infiltration in the lamina propria of the Control, LPS, and VE groups. **(B)** AB-PAS staining of colon tissue sections: panoramic images (scale bar = 625 μm) presenting the overall distribution of colon mucosal structure, and partial magnified images (scale bar = 100 μm) indicating goblet cell numbers and mucin secretion in the Control, LPS, and VE groups. **p < 0.01, ***p < 0.001,****p < 0.0001.

HE staining and AB–PAS staining of colon tissue showed that the control group had intact colonic mucosal epithelium, regular gland arrangement, and minimal inflammatory cell infiltration in the lamina propria. The LPS model group exhibited mucosal epithelial detachment, glandular atrophy and distortion, extensive inflammatory cell infiltration, reduced goblet cell numbers, and diminished mucin secretion, indicating intestinal inflammatory injury and barrier dysfunction, and leading to a significant increase in the score (**Figure 2B**). Vitamin E treatment significantly improved these pathological changes, restoring mucosal epithelial integrity, normalizing gland architecture, reducing inflammatory cell infiltration, increasing goblet cell numbers, and partially recovering mucin secretion (**Figures 3A, B**), with the Chiu’s score markedly decreasing.

**Figure 4:**
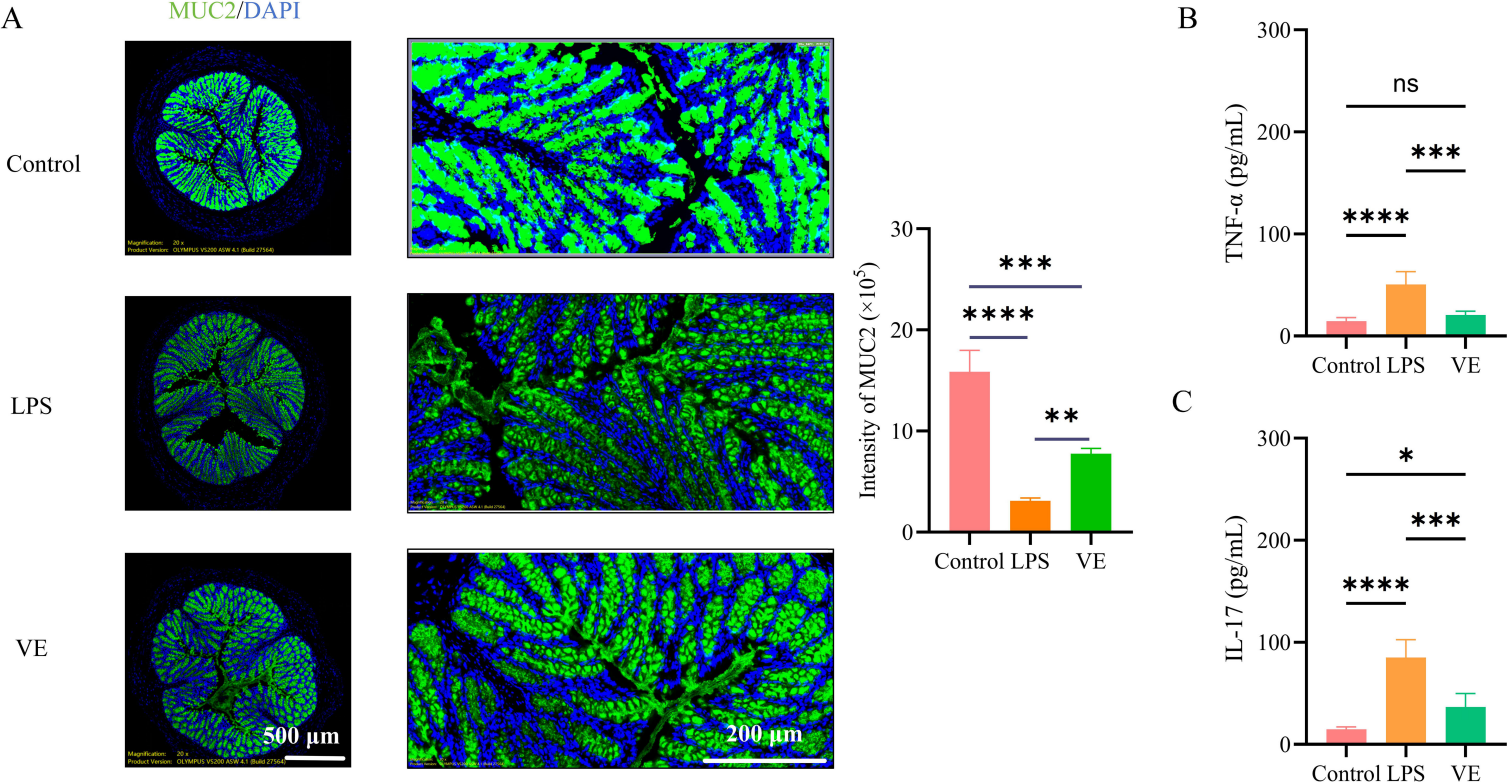
Vitamin E restores intestinal mucosal barrier function and suppresses intestinal inflammation. **(A)** Immunofluorescence staining and statistical analysis of MUC2 (green) and DAPI (blue, nuclear staining) in colon tissue: panoramic images (scale bar = 500 μm) showing the overall continuity of the MUC2-positive mucus layer in the Control, LPS, and VE groups, and partial magnified images (scale bar = 200 μm) displaying the signal intensity of MUC2 and nuclear localization (DAPI) in the three groups. **(B, C)** Bar graphs showing TNF-α **(B)** and IL-17 **(C)** levels in colon tissue detected by ELISA. Data are represented as mean ± SD. **p < 0.01, ***p < 0.001, ****p < 0.0001.

MUC2 immunofluorescence analysis revealed a continuous and thick MUC2-positive mucus layer with regularly distributed goblet cells in the control group. The LPS model group showed reduced MUC2 immunostaining intensity and fragmented mucus coverage, while Vitamin E treatment restored MUC2 signal intensity and spatial continuity (**Figure 4A**). Consistent with histological findings, Vitamin E administration reduced colonic tissue TNF-α and IL-17 levels (**Figures 4B, C**), confirming restoration of the intestinal mucus barrier and suppression of intestinal inflammation.

### 3.4 Regulation of immune cell populations by vitamin E in the lung–gut axis

**Figure 5:**
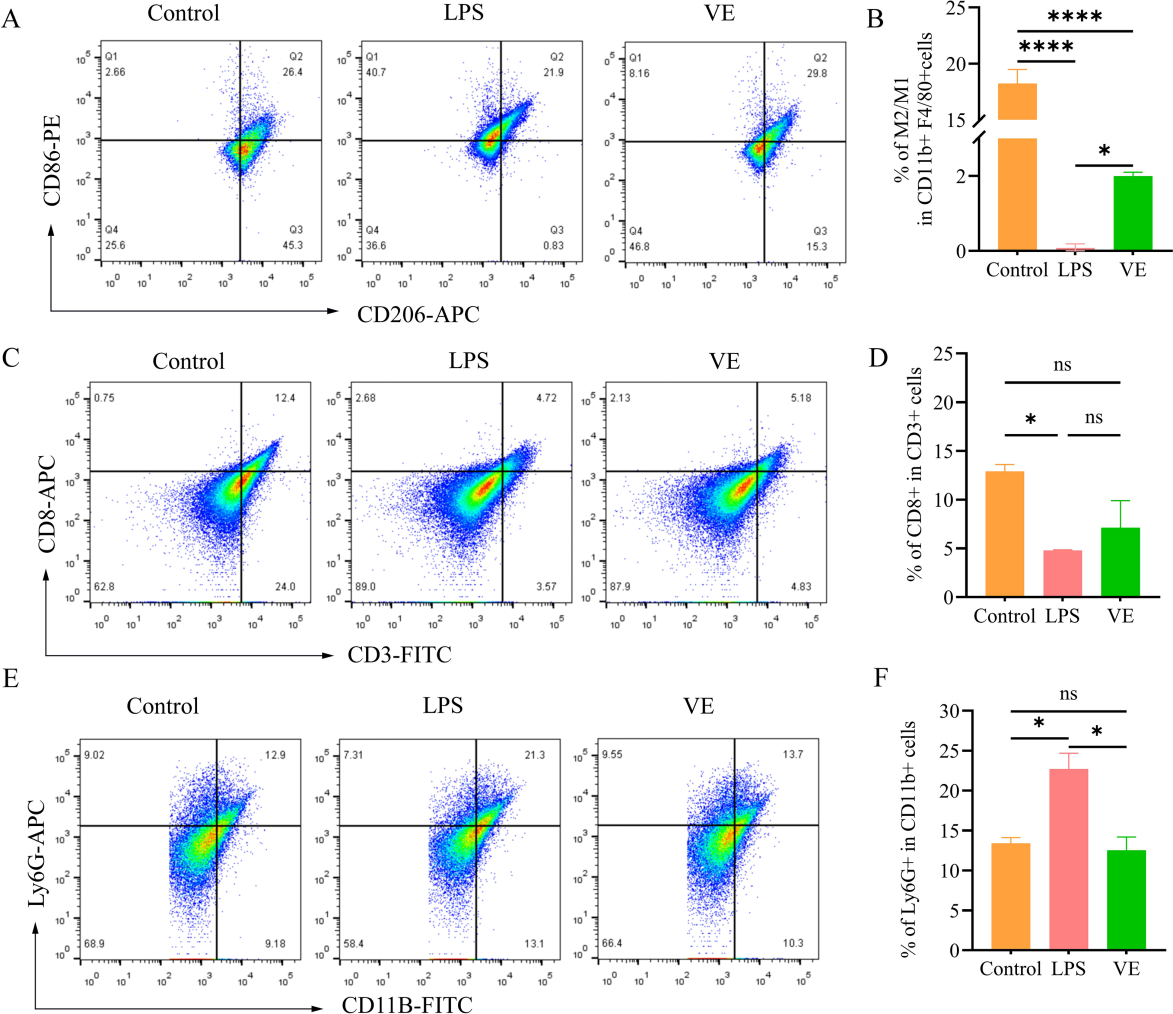
Vitamin E modulates immune cell populations in the lung–gut axis. **(A-F)** Flow cytometric analysis of immune cell subsets: **(A, B)** Lung macrophages: dot plots **(A)** and statistical graphs **(B)** depict the proportions of M1-type macrophages (CD86^+^CD206^-^) and M2-type macrophages (CD206^+^) in the Control, LPS, and VE groups; **(C, D)** Colonic cytotoxic T cells (CD3^+^CD8^+^): dot plots **(C)** and statistical graph **(D)** show the proportion of CD8^+^ T cells in each group; **(E, F)** Colonic neutrophils (Ly6G^+^CD11b^+^): dot plots **(E)** and statistical graph **(F)** illustrate the proportion of neutrophils in each group. Data are presented as mean ± SD. *ns*: no significance; *p < 0.05, ****p < 0.0001.

To further evaluate the systemic immunomodulatory effects of Vitamin E, we quantified macrophage polarization in the lung tissue. Following established protocols for characterizing macrophage balance in LPS-induced injury models (8), CD86 and CD206 were selected as representative markers for M1 and M2 phenotypes, respectively. Flow cytometric analysis showed that in lung tissue, the LPS model group had a significantly higher proportion of M1-type macrophages (CD86^+^CD206^-^) and a lower proportion of M2-type macrophages (CD206^+^) compared with the control group. Vitamin E treatment reduced M1-type macrophage proportion and increased M2-type macrophage proportion, restoring macrophage polarization balance (**Figures 5A, B**). It is important to acknowledge that M2 macrophages exhibit functional heterogeneity, including M2a, M2b, and M2c subtypes. While CD206 is a commonly used marker, its upregulation must be interpreted in conjunction with cytokine profiles. Given that M2b macrophages are characterized by high TNF-α production, the significant reduction in TNF-α observed in both lung and colon tissues of the VE-treated group supports a polarization shift toward pro-resolution M2a/M2c phenotypes rather than the M2b subtype. Furthermore, the concurrent downregulation of IL-17 and restoration of the MUC2 barrier reinforces the role of vitamin E in promoting a regulatory immune microenvironment within the lung-gut axis. In colonic tissue, the LPS model group exhibited a significantly lower proportion of cytotoxic T cells (CD3^+^CD8^+^) and a higher proportion of neutrophils (Ly6G^+^CD11b^+^) compared with the control group. Vitamin E intervention partially recovered CD8^+^ T cell proportion and significantly reduced neutrophil infiltration (**Figures 5C–F**).

## 4 Discussion

The present study systematically explored the molecular crosstalk between ALI and enteritis, and validated Vitamin E’s multi-organ protective effects using an LPS-induced ALI mouse model, providing novel insights into the “lung–intestine axis” and Vitamin E’s therapeutic potential.

The identification of 1284 shared genes between ALI and enteritis confirmed extensive molecular commonalities underlying these two diseases. This finding supported the existence of the “lung–intestine axis” at the genetic level, which was consistent with previous genetic cross-trait analysis showing significant genetic correlations between lung and gastrointestinal diseases (12). These two organs exhibit significant shared genetic susceptibility and overlapping inflammatory pathways. Therefore, the synchronous pathological changes observed in our study may reflect a coordinated response mediated by these shared systemic nodes, rather than independent, unrelated activities. These shared genes were enriched in inflammation and barrier dysfunction, and these pathways were well-recognized core pathological mechanisms of both ALI and enteritis. The 34 “ALI-Enteritis ∩ Vitamin E related” core genes served as key molecular targets bridging disease pathogenesis and Vitamin E’s action. They were involved in TNF and IL-17 signaling pathways, and these pathways were closely secondary with inflammatory response amplification and barrier function regulation (13). This finding aligned with previous studies showing that these pathways play pivotal roles in ALI and intestinal inflammation, and it provided a theoretical basis for Vitamin E to simultaneously target both diseases. The Hub Genes identified in the PPI network were likely to be critical regulatory nodes in these pathways, and Vitamin E’s modulation of these nodes might explain the compound’s multi-organ protective effects.

*In vivo* experiments confirmed that LPS-induced ALI was accompanied by secondary intestinal injury. Its characteristics included mucosal barrier dysfunction, increased inflammatory cytokine levels, and immune cell dysregulation, providing phenotypic evidence that is highly consistent with the “lung–gut axis” hypothesis within a systemic inflammatory context. Vitamin E significantly ameliorated ALI pathology by reducing pulmonary edema, inflammatory cell infiltration, and TNF-α production. The results were consistent with Vitamin E’s known anti-inflammatory properties, as demonstrated in previous studies showing that Vitamin E could suppress LPS-induced activation of alveolar macrophages (14). More importantly, Vitamin E also improved LPS-induced intestinal injury. It restored the mucosal barrier, as evidenced by increased goblet cells, mucin secretion, and MUC2 expression, and it suppressed intestinal inflammation by reducing TNF-α and IL-17 levels. This multi-organ protective effect was particularly notable, as it validated the clinical relevance of targeting shared molecular pathways between ALI and enteritis.

Immune dysregulation was a key feature of the “lung–intestine axis,” and our findings highlighted Vitamin E’s ability to modulate immune cell populations in both organs. While macrophage polarization is a complex process involving multiple markers and functional states, our selection of CD86 (M1) and CD206 (M2) was based on their established roles as classical phenotypic markers for assessing the global M1/M2 balance in acute inflammatory models. Macrophage polarization imbalance with M1 dominance in the lung was a critical driver of ALI progression. This was because M1-type macrophages secreted pro-inflammatory cytokines such as TNF-α to amplify lung injury. Macrophage polarization was a highly plastic process regulated by microenvironmental signals, and the imbalance between M1 and M2 phenotypes had been implicated in various inflammatory diseases including intestinal disorders. Vitamin E’s restoration of M1/M2 balance suggested it might shift the inflammatory microenvironment toward resolution, thereby alleviating lung damage. While Th17 cells and their IL-17 axis are recognized as major drivers of enteritis—consistent with the IL-17 downregulation observed in our study—we also identified a critical role for CD8^+^ T cells in this model of secondary intestinal injury. In the intestine, CD8^+^ T cells were essential for maintaining mucosal immune barrier function by clearing pathogens and abnormal cells. Previous studies had shown that dysfunction of CD8^+^ T cells could disrupt intestinal barrier integrity (17), which further exacerbated inflammatory responses. Neutrophil over-infiltration exacerbated intestinal mucosal injury through protease release and inflammatory mediator secretion. Vitamin E’s recovery of CD8^+^ T cell proportion and suppression of neutrophil infiltration further supported its role in regulating intestinal immune homeostasis. This function was crucial for preventing secondary enteritis following ALI. Consequently, Vitamin E exerts a dual protective effect: it suppresses IL-17-mediated inflammation while rescuing the CD8^+^ T cell population, thereby restoring both the immunological and physical integrity of the intestinal barrier.

Although Vitamin E is a well-established pharmacological agent, our study leverages network pharmacology to uncover its novel application within the emerging framework of the ‘lung–gut axis.’ It is worth noting that the sustained improvement effect of Vitamin E on pathological conditions of the lungs and intestines is consistent with the predicted results obtained through network pharmacology. This study not only confirmed that the core genes (the intersection of ALI-Enteritis and Vitamin E related genes) and their downstream pathways, including TNF and IL-17, are functional targets of Vitamin E, thereby providing a mechanistic framework for its cross-organ regulatory effects and strengthening the study’s internal validity, but also highlighted, from a holistic systems level perspective, the value of drug repurposing in treating complex systemic inflammatory syndromes. However, several limitations should be acknowledged. First, the specific Hub Genes mediating Vitamin E’s effects were not individually validated. Future studies should focus on gene silencing or overexpression to confirm their causal roles. Second, the study only explored the LPS-induced model. While our findings are based on a single ALI model, the core mechanisms identified are likely conserved across etiologies, whether Vitamin E exerts similar effects in ALI caused by other etiologies, such as viral, sepsis and trauma, required further investigation. Thirdly, while our data demonstrate parallel therapeutic effects of Vitamin E in both organs, they do not provide direct evidence of causality or the specific route of lung-to-gut crosstalk. Future studies employing adoptive cell transfer or parabiotic models are needed to definitively characterize the directional signaling within this axis.

## 5 Conclusion

This study combined network pharmacology with *in vivo* validation to demonstrate regulatory effects of Vitamin E on the ALI-intestinal inflammation axis that are consistent with the suppression of TNF and IL-17 pathways. At the network level, ALI and intestinal inflammation shared 1284 genes, and their intersection with Vitamin E identified 34 key targets. PPI and enrichment analyses revealed core pathways including inflammation, TNF and IL-17 signaling, suggesting Vitamin E might restore systemic immune homeostasis through multi-target synergy. *In vivo*, LPS nasal instillation established an ALI model, where Vitamin E significantly alleviated alveolar inflammation, mitigated pulmonary edema, and downregulated TNF-α. More importantly, Vitamin E repaired the intestinal mucosal barrier, restored the MUC2 mucus layer, reduced IL-17 levels and neutrophil infiltration, achieving synchronous intervention on the lung-intestinal axis. Mechanistically, as a lipid-soluble antioxidant, Vitamin E modulated macrophage polarization and neutrophil migration to attenuate inflammation amplification, thereby disrupting the lung injury-intestinal inflammation cycle. This study, spanning systems biology to organ interaction, proposed that Vitamin E improved ALI and secondary intestinal inflammation via multi-level mechanisms: shared genes, immune microenvironment regulation, and barrier function restoration, offering insights and an experimental basis for managing multi-organ injuries through nutritional immune intervention.

## Data Availability

The original contributions presented in the study are included in the article/**Supplementary Material**. Further inquiries can be directed to the corresponding authors.

## Supplementary material

Supplementary Figure 1 Gating strategy. (A-C) Gating strategies of neutrophils, TAM cells and T cells.

## References

[1] Matute-Bello G Frevert CW Martin TR . Animal models of acute lung injury. Am J Physiol Lung Cell Mol Physiol. (2008) 295:L379–99. doi: 10.1152/ajplung.00010.2008, PMID: 18621912 PMC2536793

[2] Sencio V Barthelemy A Tavares LP Machado MG Soulard D Cuinat C . Gut dysbiosis during influenza contributes to pulmonary pneumococcal superinfection through altered short-chain fatty acid production. Cell Rep. (2020) 30:2934–2947.e6. doi: 10.1016/j.celrep.2020.02.013, PMID: 32130898

[3] Budden KF Gellatly SL Wood DL Cooper MA Morrison M Hugenholtz P . Emerging pathogenic links between microbiota and the gut-lung axis. Nat Rev Microbiol. (2017) 15:55–63. doi: 10.1038/nrmicro.2016.142, PMID: 27694885

[4] Baden LR Drazen JM Kritek PA Curfman GD Morrissey S Campion EW . H1N1 influenza A disease--information for health professionals. N Engl J Med. (2009) 360:2666–7. doi: 10.1056/NEJMe0903992, PMID: 19423873

[5] Wang J Li F Wei H Lian ZX Sun R Tian Z . Respiratory influenza virus infection induces intestinal immune injury via microbiota-mediated Th17 cell-dependent inflammation. J Exp Med. (2014) 211:2397–410. doi: 10.1084/jem.20140625, PMID: 25366965 PMC4235643

[6] Heller R Werner-Felmayer G Werner ER . Alpha-tocopherol and endothelial nitric oxide synthesis. Ann N Y Acad Sci. (2004) 1031:74–85. doi: 10.1196/annals.1331.007, PMID: 15753135

[7] Brigelius-Flohé R Traber MG . Vitamin E: function and metabolism. FASEB J. (1999) 13:1145–55. doi: 10.1096/fasebj.13.10.1145, PMID: 10385606

[8] Pan J Li Z Zhu M Guo L Chen W Yu L . Vitamin E exerts a mitigating effect on LPS-induced acute lung injury by regulating macrophage polarization through the AMPK/NRF2/NF-κB pathway. Int Immunopharmacol. (2025) 159:114893. doi: 10.1016/j.intimp.2025.114893, PMID: 40403505

[9] Fan X Yin J Yin J Weng X Ding R . Comparison of the anti-inflammatory effects of vitamin E and vitamin D on a rat model of dextran sulfate sodium-induced ulcerative colitis. Exp Ther Med. (2023) 25:98. doi: 10.3892/etm.2023.11797, PMID: 36761001 PMC9893224

[10] Aguirre-Plans J Piñero J Menche J Sanz F Furlong LI Schmidt HHHW . Proximal pathway enrichment analysis for targeting comorbid diseases via network endopharmacology. Pharm (Basel). (2018) 11:61. doi: 10.3390/ph11030061, PMID: 29932108 PMC6160959

[11] Chiu CJ Mcardle AH Brown R Scott HJ Gurd FN . Intestinal mucosal lesion in low-flow states. i. a morphological, hemodynamic, and metabolic reappraisal. Arch Surg. (1970) 101:478–83. doi: 10.1001/archsurg.1970.01340280030009, PMID: 5457245

[12] You D Wu Y Lu M Shao F Tang Y Liu S . A genome-wide cross-trait analysis characterizes the shared genetic architecture between lung and gastrointestinal diseases. Nat Commun. (2025) 16:3032. doi: 10.1038/s41467-025-58248-w, PMID: 40155373 PMC11953465

[13] Shale M Ghosh S . Beyond TNF, Th1 and Th2 in inflammatory bowel disease. Gut. (2008) 57:1349–51. doi: 10.1136/gut.2008.151563, PMID: 18791115

[14] Pathania V Syal N Pathak CM Khanduja KL . Vitamin E suppresses the induction of reactive oxygen species release by lipopolysaccharide, interleukin-1beta and tumor necrosis factor-alpha in rat alveolar macrophages. J Nutr Sci Vitaminol (Tokyo). (1999) 45:675–86. doi: 10.3177/jnsv.45.675, PMID: 10737222

[15] HouF XiaoJ WangH XiaoK YangW ZhaoD . Alveolar macrophage-derived TGF-β promotes acute lung injury recovery by regulating inflammatory monocyte-derived macrophages. J Adv Res. (2025) S2090-1232(25)00869-0. doi: 10.1016/j.jare.2025.10.075, PMID: 41207507

[16] Zhang M Li X Zhang Q Yang J Liu G . Roles of macrophages on ulcerative colitis and colitis-associated colorectal cancer. Front Immunol. (2023) 14:1103617. doi: 10.3389/fimmu.2023.1103617, PMID: 37006260 PMC10062481

[17] Das Adhikari U Froehle LM Pipkin AN Baharlou H Linder AH Shah P . Immunometabolic defects of CD8+ T cells disrupt gut barrier integrity in people with HIV. Cell. (2025) 188:5666–5679.e19. 40939591 10.1016/j.cell.2025.08.024 PMC12641501

