# Supplementary material for: Integrated network pharmacology and in vivo evidence reveal vitamin E’s multi-organ protective effects in acute lung injury and secondary enteritis
Source: Front Immunol. 2026 Mar 18;17:1762374. doi: 10.3389/fimmu.2026.1762374 (PMC13038555; doi:10.3389/fimmu.2026.1762374)
Supplement: Supplementary file 1 [file DataSheet1.pdf]

frontiers

The International Society for the Advancement of Cytometry (ISAC) has highlighted the importance of including comprehensive methodological information to ensure data reproducibility and reliability. In line with this, Frontiers in Immunology now requires authors to submit a checklist for manuscripts that involve flow or mass cytometry. This checklist helps standardize the reporting process, improving the quality and transparency of published data. By doing so, we support scientific progress, making it easier for other researchers to replicate and validate experiments.

This form should be submitted with any manuscripts using flow or mass cytometry.

Sample/specimen/material description

- [ ] Total blood
- [ ] PBMCs
- [x] Organ digests

Other Lung and colon

Did the samples suffer any treatment before or after incubation with the antibodies?

- [x] Drug LPS/Vitamin E/Saline
- [ ] Cell permeabilization ______________________
- [ ] Dye ______________________________________
- [ ] Propidium iodine
- [ ] Not applicable

Other ____

Instrument and antibodies

Name of the Cytometer BD LSRFortessa

Antibodies and targets	Fluorochrome/ Metal	Catalog number/Company
e.g. anti-CD4	FITC	Cat. XXX/ XXX ltd.
anti-CD11b	FITC	BioLegend

anti-F4/80	PE/Cyanine7	BioLegend
anti-CD86	PE	BioLegend
anti-CD206	APC	BioLegend
anti-CD3	FITC	BioLegend
anti-CD8	APC	BioLegend
anti-Ly6G	APC	BioLegend

Data analyses

1. Name of the software FlowJo-V10________________________________
2. Reference gating strategy in the manuscript or supplementary material

Gating strategy in (eg Figure X) Figure 5_____________________________

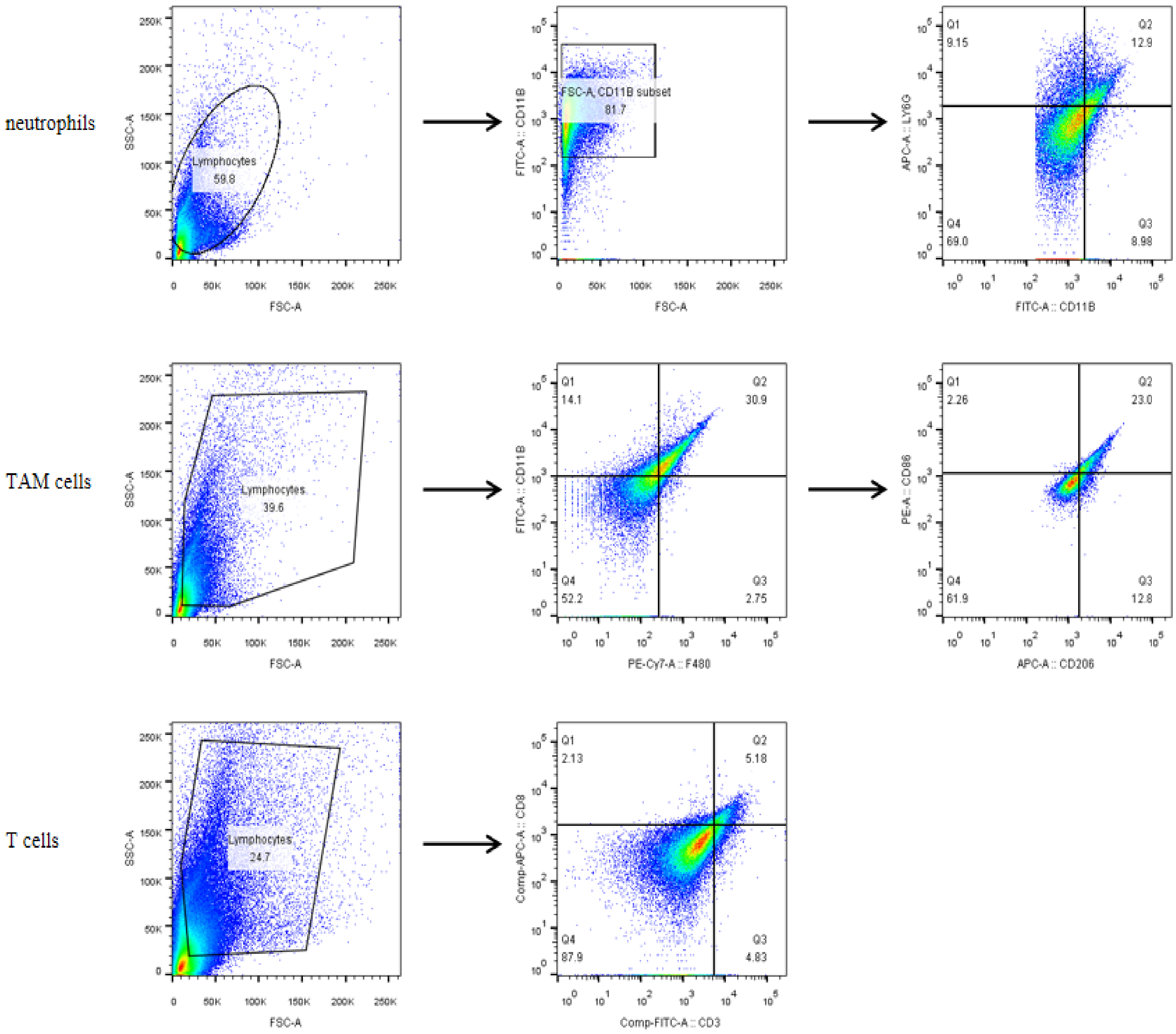

neutrophils
Lymphocytes
59.8
SSC-A
FSC-A
FSC-A, CD11B subset
81.7
FITC-A :: CD11B
FSC-A
Q1
9.15
Q2
12.9
Q4
69.0
Q3
8.98
APC-A :: LY6G
FITC-A :: CD11B
TAM cells
Lymphocytes
39.6
SSC-A
FSC-A
Q1
14.1
Q2
30.9
Q4
52.2
Q3
2.75
FITC-A :: CD11B
PE-Cy7-A :: F480
Q1
2.26
Q2
23.0
Q4
61.9
Q3
12.8
PE-A :: CD86
APC-A :: CD206
T cells
Lymphocytes
24.7
SSC-A
FSC-A
Q1
2.13
Q2
5.18
Q4
87.9
Q3
4.83
Comp-APC-A :: CD8
Comp-FITC-A :: CD3
