# Supplementary figures and images for: Integrated network pharmacology and in vivo evidence reveal vitamin E’s multi-organ protective effects in acute lung injury and secondary enteritis
Source: Front Immunol. 2026 Mar 18;17:1762374. doi: 10.3389/fimmu.2026.1762374 (PMC13038555; doi:10.3389/fimmu.2026.1762374)

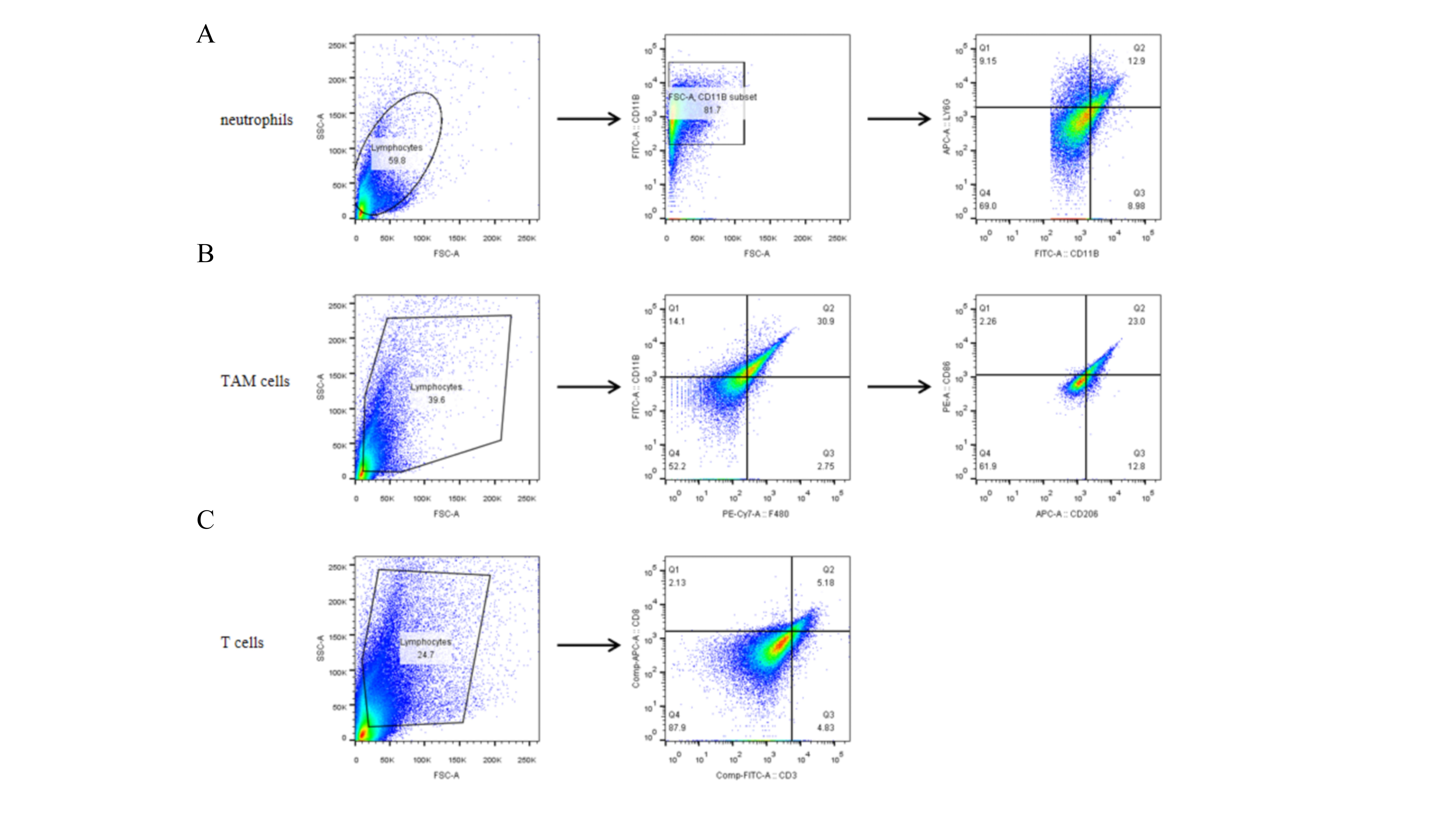

Supplement: Supplementary Figure 1 — Gating strategy. (A-C) Gating strategies of neutrophils, TAM cells and T cells. [file Image1.jpeg]
